# Interacting Effects of Discharge and Channel Morphology on Transport of Semibuoyant Fish Eggs in Large, Altered River Systems

**DOI:** 10.1371/journal.pone.0096599

**Published:** 2014-05-06

**Authors:** Thomas A. Worthington, Shannon K. Brewer, Nicole Farless, Timothy B. Grabowski, Mark S. Gregory

**Affiliations:** 1 Oklahoma Cooperative Fish and Wildlife Research Unit, Oklahoma State University, Stillwater, Oklahoma, United States of America; 2 U.S. Geological Survey, Oklahoma Cooperative Fish and Wildlife Research Unit, Oklahoma State University, Stillwater, Oklahoma, United States of America; 3 U.S. Geological Survey, Texas Cooperative Fish and Wildlife Research Unit, Texas Tech University, Lubbock, Texas, United States of America; 4 Natural Resource Ecology and Management, Oklahoma State University, Stillwater, Oklahoma, United States of America; Pacific Northwest National Laboratory, United States of America

## Abstract

Habitat fragmentation and flow regulation are significant factors related to the decline and extinction of freshwater biota. Pelagic-broadcast spawning cyprinids require moving water and some length of unfragmented stream to complete their life cycle. However, it is unknown how discharge and habitat features interact at multiple spatial scales to alter the transport of semi-buoyant fish eggs. Our objective was to assess the relationship between downstream drift of semi-buoyant egg surrogates (gellan beads) and discharge and habitat complexity. We quantified transport time of a known quantity of beads using 2–3 sampling devices at each of seven locations on the North Canadian and Canadian rivers. Transport time was assessed based on median capture time (time at which 50% of beads were captured) and sampling period (time period when 2.5% and 97.5% of beads were captured). Habitat complexity was assessed by calculating width∶depth ratios at each site, and several habitat metrics determined using analyses of aerial photographs. Median time of egg capture was negatively correlated to site discharge. The temporal extent of the sampling period at each site was negatively correlated to both site discharge and habitat-patch dispersion. Our results highlight the role of discharge in driving transport times, but also indicate that higher dispersion of habitat patches relates to increased retention of beads within the river. These results could be used to target restoration activities or prioritize water use to create and maintain habitat complexity within large, fragmented river systems.

## Introduction

Habitat fragmentation, loss and degradation are frequently cited as main causes of species decline and extinction across all biomes [1]–[3]. Within the United States, 85% of large rivers are impacted by the presence of barriers [4], resulting in the natural continuum being divided into poorly connected fragments [5]. This degradation of natural habitat, alongside other factors, has resulted in approximately 40% of fish species in continental North American being considered imperilled [6]. In the Great Plains, there has been a marked decline in the native fish fauna over the preceding 50 years, with reductions in both distribution and abundance of many species that exhibit unique life-history adaptations [7].

Under natural conditions, the rivers of the Great Plains are subject to extremes in physicochemical conditions [8], with the timing of high and low-flow events subject to extensive temporal variability [9], [10]. In response to such conditions, aquatic organisms may undertake bet-hedging strategies as part of their life history [11]. Pelagic-broadcast spawning cyprinids (pelagophils) are a reproductive guild of small minnows [12]. These species were historically widespread and abundant in rivers of the Great Plains but many have undergone dramatic declines and extirpations [13]–[16]. These species, which include the federally threatened Arkansas River shiner (*Notropis girardi*) produce eggs that achieve semi-buoyancy soon after fertilization, but require water movement to remain in suspension [12], [17]. Members of this reproductive guild also display fractional or extended spawning [18]–[23], a potential mechanism to cope with discharge variability. Eggs and larvae may remain in suspension for 3–5 days before they reach a free-swimming stage [24], [25], and therefore potentially require extensive sections of free-flowing river [5], [26], [27].

The life history of pelagophils renders them particularly sensitive to river fragmentation [28]. The construction of large reservoirs throughout the Great Plains has dramatically altered the natural flow regime [29], [30]. Reproductive success for pelagic-broadcast spawning cyprinids is thought to be intrinsically linked to flow availability and magnitude [31]. Spawning may take place at any point during the extended reproductive period, with an increase in reproduction in response to high flow events [21], [22], [32], [33]. However, reproductive success is thought to be non-existent during period of no flow [21], [32]. The reduction in the length of unfragmented channel sections may result in the ichthyoplankton being washed into unsuitable habitats, such as reservoirs, where they risk being smothered in sediment or subjected to increased predation risk [12]. It has been proposed that pelagic-broadcast spawners must undertake upstream migrations to counteract downstream drift [12], [14], [34] but these movements would be truncated by the presence of dams [35].

Several studies have examined egg transport at catchment scales for Great Plains pelagic-broadcast spawning cyprinids e.g., [36]–[38]; however, little research has quantified the role of channel complexity at the reach scale on the downstream movement of semi-buoyant fish eggs. The aim of this study was to examine how discharge and habitat heterogeneity impact the movement rate of egg surrogates in two rivers within the historical distribution of Arkansas River shiner [16]. We hypothesized that downstream drift of egg surrogates would be controlled by the interaction of the flow regime and river geomorphology, with drift rate reduced in reaches with greater habitat complexity.

## Materials and Methods

### Ethics Statement

Work in this study took place under a state-wide Oklahoma Department of Wildlife Conservation, Scientific Collector Special License. In case of accidental captures of eggs or individuals of the federally listed Arkansas River shiner, an endangered species collection permit was obtained from the U.S. Fish and Wildlife Service covering the Canadian River basin. Where necessary while working on private land, landowner permission was sought to access the river. No endangered or protected species were involved in this study.

### Habitat Complexity

**Figure 1:**
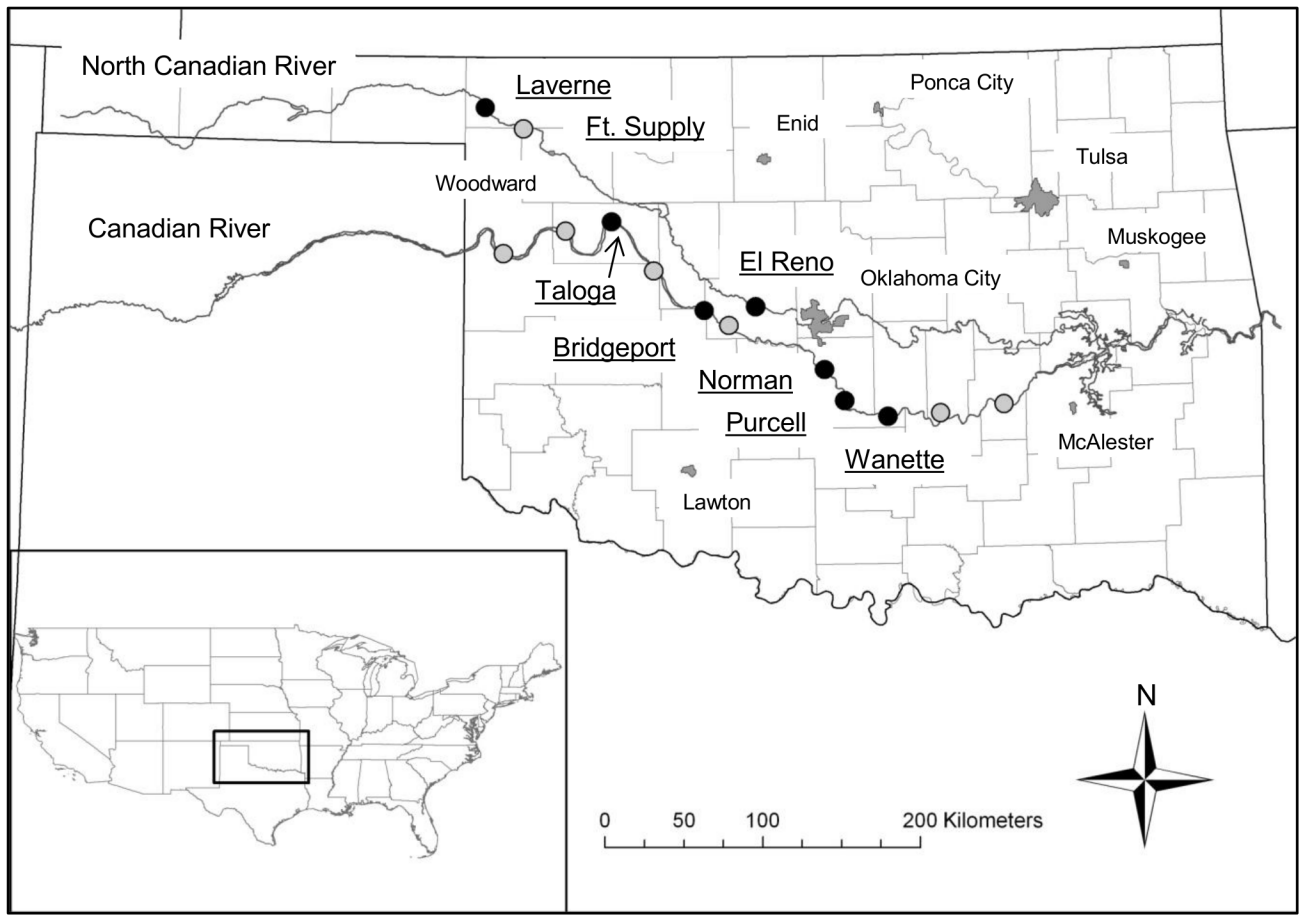
Location of U.S. Fish and Wildlife Service fisheries survey sites, plus replacement site. Study area showing the Canadian and North Canadian Rivers. Sites assessed for habitat metrics only (grey circles) and those used in the egg transport trials (black circles) Major urban areas in Oklahoma labelled.

Experiments were completed on the Canadian and North Canadian rivers, Oklahoma to assess the relation between habitat complexity and egg transport time. Prior to field sampling, habitat structure of 13 potential sites was assessed using FRAGSTATS version 4.0 [39]. Thirteen sites were selected from U.S. Fish and Wildlife Service survey locations and were reaches where Arkansas River shiner was present (Canadian River, n = 11) and absent (North Canadian River, n = 2) from surveys conducted in 2007–2009 (D. Fenner, personal communication; Figure 1).

**Figure 2:**
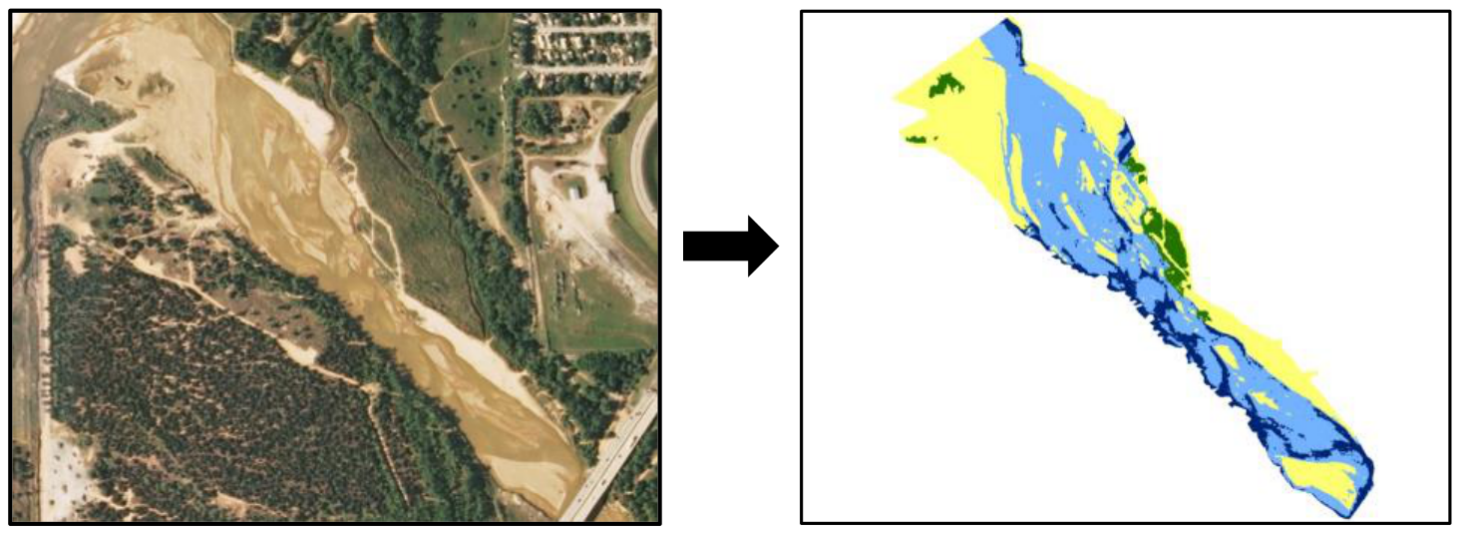
Conversion of aerial photograph into a raster containing four habitat classes. Habitat classes: green, vegetation; light blue, shallow water; dark blue, deep water and yellow, sand.

We downloaded the most recent aerial photographs (2010) of the thirteen sites (Geospatial Data Gateway; http://datagateway.nrcs.usda.gov/GDGHome.aspx), and displayed them in ArcMap 9.3 (ESRI, Redlands, CA, USA). A 1000-m section of the channel upstream of each survey site was selected and the boundary of the channel, marked by a continuous vegetation edge, was delineated. This polygon was used to clip the area of interest from band 1 of the aerial photograph. The reclassify tool in ArcGIS was used to create a new raster layer by splitting band 1 into four habitat categories representing deep water (sand not visible), shallow water (sand visible), exposed sand, and vegetation (Figure 2). The newly created layer was visually inspected for consistency with the original aerial photograph and areas of incongruence were manually corrected.

We used FRAGSTATS to calculate the area of each habitat category as a percentage of the overall landscape, and three landscape metrics: (1) total landscape area, (2) mean ‘shape index’ (the complexity of patch shapes in comparison to a square) and (3) mean ‘contagion index’ (level to which patches were dispersed across the landscape; [40]). Human dominated landscapes (e.g. urban and agriculture) tend to have lower patch shape complexity than those less influenced by anthropogenic activities [40], [41]. Conversely, an examination of three Swiss rivers found reduced mean shape index in more natural reaches, which equated to a reduced edge effect [42]. Habitat patch shape can influence a number of ecological processes, including nest site selection and reproductive success [43], emigration [44], and colonization [45]. The spatial configuration (i.e. contagion) of habitat patches helps to determine landscape connectivity [46], with highly fragmented landscapes having lower contagion scores. In the context of this study, a lower contagion index would suggest an intermixed sand/water landscape as expected in the braided channels of Great Plains rivers, rather than a homogenized profile in more managed reaches.

After calculating the landscape metrics described above, sites were ranked based on these metrics to provide an overall score of habitat complexity and integrity (Table 1). Five sites on the Canadian River (36°3′14″N, 98°58′8″W to 34°55′11″N, 97°2′58″W) were selected to represent a range of the habitat complexity and integrity scores, whereas both sites from the North Canadian River, were selected due to the species being absent from recent surveys on that river. One site (Ft. Supply) was not useable due to density of vegetation in the channel and was replaced with another site on the North Canadian River (El Reno; 36°42′35″N, 99°50′59″W to 35°33′45″N, 97°57′33″W). One site on the South Canadian (Norman) was moved ∼1 km downstream due to difficulties obtaining access. Habitat complexity was quantified over a 1000-m section for both replacement sites using the same FRAGSTATS procedure described above.

**Table 1:** FRAGSTATS metrics calculated for thirteen US Fish and Wildlife Service fisheries survey sites on the Canadian and North Canadian rivers and the two replacement sites (not ranked).

Site	River	Total Area (km^2^)	% Vegetation	% Shallow Water	% Deep Water	% Sand	Shape Index	Contagion Index	Average Rank#
Caddo	Canadian	6.53 (8)	0.00	32.20	47.68	20.12	1.49 (2)	38.54 (2)	4
**Wanette**	**Canadian**	**14.86 (3)**	**0.00**	**24.75**	**35.18**	**40.07**	**1.41 (6)**	**40.51 (3)**	**4**
Roll	Canadian	6.03 (9)	0.00	45.81	18.61	35.59	1.38 (7)	38.4 (1)	5.67
**Bridgeport**	**Canadian**	**8.08 (7)**	**0.06**	**10.76**	**40.26**	**48.92**	**1.61 (1)**	**58.44 (12)**	**6.67**
Ada	Canadian	11.57 (5)	0.00	37.43	9.00	53.57	1.34 (13)	42.63 (4)	7.33
**Taloga**	**Canadian**	**5.68 (10)**	**1.24**	**37.31**	**15.35**	**46.10**	**1.48 (3)**	**49.03 (9)**	**7.33**
**Ft. Supply**	**North Canadian**	**0.69 (13)**	**1.40**	**46.22**	**52.38**	**0.00**	**1.47 (4)**	**45.74 (5)**	**7.33**
Calvin	Canadian	17.98 (1)	0.00	20.42	7.20	72.38	1.37 (9)	59.35 (13)	7.67
**Norman**	**Canadian**	**16.56 (2)**	**4.25**	**44.99**	**11.09**	**39.68**	**1.36 (11)**	**51.76 (10)**	**7.67**
Thomas	Canadian	9.29 (6)	0.00	49.42	5.69	44.89	1.37 (10)	47.69 (7)	7.67
**Laverne**	**North Canadian**	**0.69 (12)**	**3.42**	**36.10**	**54.14**	**6.34**	**1.42 (5)**	**46.5 (6)**	**7.67**
Camargo	Canadian	4.47 (11)	3.10	51.94	23.75	21.21	1.37 (8)	48.4 (8)	9
**Purcell**	**Canadian**	**12.03 (4)**	**1.41**	**45.96**	**9.99**	**42.65**	**1.35 (12)**	**54.32 (11)**	**9**
Norman	Canadian	13.79	7.92	23.20	20.73	48.14	1.44	46.82	
El Reno	North Canadian	2.15	8.54	7.18	82.46	1.82	1.33	67.66	

# Initial sites ranked on the metrics total area, shape index and contagion index. Number in parentheses shows ranking. Sites in bold represent those selected for egg transport tests.

As the sand bed rivers of the Great Plains can exhibit temporal variation in physical structure, we field validated the accuracy of aerial photography habitat classifications at each site. Inaccuracy in the habitat classifications has the potential to introduce bias into some of the explanatory variables used in our analysis. To assess this we randomly generated 25 points using ArcGIS and navigated to each of these points using a Trimble GeoXH differential global positioning system (DGPS; sub-meter accuracy; Trimble Navigation Limited, Sunnyvale, California, USA). We assessed habitat classification at each point: sand, water (shallow and deep combined) or vegetation. At our replacement site on the North Canadian River, we haphazardly chose 25 points and classified the habitat, which we later verified using the aerial photograph. We compared our habitat classification using aerial photographs against field measurements to determine accuracy.

### Egg Transport Time

Transport time was assessed by releasing a known quantity of egg surrogates (gellan beads) and recording the temporal distribution of recaptures. Gellan beads (Technology Flavors and Fragrances, Inc., Amityville, New York) have similar physical properties including shape and specific gravity to the eggs of pelagic-broadcast spawning cyprinids [37], [38]. Gellan beads were soaked in freshwater for a minimum of 24 hrs prior to release [47] to more closely match the specific gravity of Arkansas River shiner eggs [17]. At each sampling site, 3,450 g of gellan beads were released, equating to approximately 100,000 (95% CI: 98,690–101,050) beads. The 95% confidence interval for the weight of a single bead was calculated by weighing 52 batches of 1,000 beads to the nearest 0.01 g. Gellan beads were rinsed before weighing to remove excess syrup used as preservative during storage.

Experiments took place in March 2013. The gellan beads were recaptured using Moore egg collectors (MECs; see [48] for a complete description). Briefly, the MEC is a device designed for the collection of semi-buoyant fish eggs. The MEC is secured facing the direction of flow, in the upper portion of the water column [49]. The open upstream end of the MEC allows floating propagules to enter the device from where they are swept up a mesh screen at the water-air interface. For the five sites initially assessed for habitat complexity, the downstream location of the MECs and the upstream release point of the gellan beads were identified using ArcGIS by delineating the middle 500-m of the 1000-m aerial photography habitat assessment section. We located the gellan bead release point (250-m from the upstream end of the habitat analysis section) and the MEC location (750-m) using a DGPS, therefore the beads travelled 500-m. For the two replacement sites, a suitable deployment location for the MECs was selected near the access to the river and the release point of the gellan beads measured 500-m upstream. At each site, two or three MECs were deployed in areas of concentrated flow as laboratory studies indicated the surrogate fish eggs were highly spatially aggregated and therefore likely to move in areas of bulk flow [48]. The MECs were placed in parallel, orthogonal to the shoreline, facing the direction of flow with the opening of the box submerged just below the waterline. The gellan beads were released upstream at single point in the area of concentrated discharge. A single observer was assigned to each MEC and the number of beads captured every minute was counted manually until no recaptures occurred for a period of 5 consecutive minutes.

We examined variability in fluvial geometry among sampling sites by measuring five width-to-depth ratios [50], at each site. Width∶depth was measured at five randomly selected transects, stratified every 100-m through the study site. Depth was measured every 1-m along each transect and the mean depth used to calculate the width∶depth ratio. The site statistic was the mean of the five individual ratios. Discharge was measured at each site using the velocity-area method at a minimum of one of the width-to-depth transects and where the river formed a single channel [50]. Discharge was also assessed using gage measurements at the time of the bead release from the closest U.S. Geological Survey (USGS) gage on the same river (Gage numbers: 07229200, 07228500, 07239500, 07237500; http://waterdata.usgs.gov/usa/nwis/rt).

### Statistical Analyses

Transport time was assessed by comparing the temporal distributions of gellan bead captures among sites. Bead captures from the individual MECs at each site were combined to produce a site measure and plotted as a cumulative distribution function. Two separate models were constructed to evaluate the median capture time (the time at which 50% of gellan beads had been captured) and the sampling period (the time period between which 2.5% to 97.5% of gellan beads were captured). Times were calculated in minutes and decimal seconds by interpolating between the two points. The relationship between median capture time of gellan beads and sampling period was assessed using Pearson product-moment correlation.

The relationship between the dependent variables, median capture time and sampling period, and the explanatory variables was modelled using ordinary least squares regression models. Initial explanatory variables were the four metrics calculated using FRAGSTATS, the mean of the site width-to-depth ratios, site discharge, and the USGS gage discharge values. We used Pearson product-moment correlation tests to assess colinearity among our explanatory variables. If significant correlations existed between variables, we used only a single variable in our model building trials. Predictor selection was carried out using a combination of a hierarchical framework based on *a priori* knowledge and forward-entry method. Discharge was entered first because we expected it to most heavily influence drift of the beads. The remaining variables were then sequentially entered into the model and improvements in model fit were assessed after each variable was entered. Predictors were retained in the model if they were significantly related to the dependent variable (α<0.05). Data on the median capture time and the sampling period were transformed (natural log) if examination of standardized residuals and Cook's distance measure [51], suggested model assumptions were violated. If assumptions were still violated, bootstrapping was used (1,000 iterations) to provide robust confidence intervals (CI) of the parameter *β* values [52]. Relative importance of the independent variables to the model fit was assessed by examining zero-order correlation for each predictor [53]. All statistical analyses were completed using SPSS (SPSS 20.0.0, IBM Corp).

## Results

### Habitat Complexity

There was considerable variation in the habitat metrics calculated via FRAGSTATS (Table 1). Site total area was particularly variable but followed a fairly consistent pattern, with total area increasing from upstream to downstream and those sites on the North Canadian having a smaller total area than those on the Canadian River. Habitat heterogeneity was highly variable among sites: Wanette had the most heterogeneous habitat, ranking high across all metrics, whereas El Reno had a low contagion index (Table 1). Field validation showed reasonable congruence among habitat categories assigned from the aerial photographs and field measurements. Across all seven sites, 73% (range: 64%–84%) of 25 points were correctly assigned to a habitat category (Table 2).

**Table 2:** Median capture time, sampling period length (in parentheses) and transport velocities of egg transport experiments on the Canadian and North Canadian rivers.

Site	Median capture time (h:mm:ss) (95% CI)	Transport velocity (m/s) (95% CI)	Mean width(m): depth(m) ratio	Site discharge (m^3^s^−1^)	Correct habitat classifications (%)
Wanette	0:17:12 (0:13:41–0:36:27) *	0.48 (0.23–0.61)	387∶1	7.50	72
Purcell	0:15:16 (0:12:15–0:25:09) *	0.55 (0.33–0.68)	151∶1	4.88	64
Norman	0:15:27 (0:12:27–0:20:14) *	0.54 (0.41–0.67)	234∶1	7.11	80
Bridgeport	0:16:13 (0:14:12–0:18:56)	0.51 (0.44–0.59)	240∶1	4.09	72
Taloga	0:20:58 (0:14:46–1:06:59) *	0.40 (0.12–0.56)	163∶1	2.73	72
El Reno	0:31:57 (0:28:28–0:37:43)	0.26 (0.22–0.29)	47∶1	1.44	68
Laverne	1:55:47 (0:55:23–4:13:07)	0.07 (0.03–0.15)	68∶1	0.04	84

Mean width∶depth ratio and site discharges used in the regression models and the number of correctly assigned habitat point classifications at each site.

* Data for these sites published in Worthington T, Brewer SK, Farless N (2013) Spatial and temporal variation in efficiency of the Moore Egg Collector. North American Journal of Fisheries Management 33: 1113–1118.

### Egg Transport

Median capture time was remarkably similar for the Canadian River sites, ranging from 15 to 21 minutes (Table 2). For the North Canadian sites, the median time to capture at the El Reno site was closer to that of the Canadian River (∼32 minutes), whereas Laverne was significantly greater (almost 2 hours, Table 2). Median capture time and sampling period were highly correlated (*r* = 0.96, *n* = 7, *P* = 0.01). Further examination suggested the relationship was overly influenced by the Laverne point. The Laverne point was removed and no relationship between median capture time and sampling period was evident (*r* = 0.06, *n* = 6, *P* = 0.90). Unlike median capture time, sampling period was far more variable ranging from 5 to 52 minutes for the Canadian River sites. Sampling period was shortest at Bridgeport with 95% of gellan beads being captured within 5 minutes, compared to approximately 200 minutes at Laverne.

Pearson product-moment correlations revealed high levels of colinearity between site discharge and width-to-depth ratio (*r* = 0.87, *n* = 7, *P* = 0.01), total site area (*r* = 0.98, *n* = 7, *P*<0.001) and USGS gage discharge (*r* = 0.95, *n* = 7, *P*<0.01). Site discharge was predicted to be the primary factor determining downstream drift, therefore this variable was selected for use in the models. The vegetation category was relatively uncommon (<9%) in the aerial photographs and was therefore removed from analysis. The percentage of water at a site (shallow and deep combined) was also omitted because it was significantly correlated with the percentage of sand (*r* = −0.96, *n* = 7, *P*<0.01). Final explanatory variables were: percentage of sand at a site, mean shape index, mean contagion index and site discharge.

Our final ordinary least squares regression models indicated median capture time was negatively related to site discharge (*F*_5, 6_ = 8.53, *P* = 0.03), with the model explaining 63% of the observed variation. The bootstrapped confidence intervals of the discharge predictor confirmed the negative relationship between discharge and median capture time *β* = −0.21 (95% CI: −0.35–−0.05). Sampling period was negatively related to two predictor variables (*F*_4, 6_ = 38.07, *P*<0.01), site discharge (*t* = −7.52, *P*<0.01) and contagion index (*t* = −7.35, *P*<0.01), with the model explaining 95% of the observed variance. Bootstrapped confident intervals confirmed the negative relationship between the predictor variables and sampling period: discharge *β* = −0.44 (95% CI: −0.57–−0.40), contagion index *β* = −0.13 (95% CI: −0.23–−0.12). Examination of the zero-order correlations for each predictor suggested discharge (−0.53) was slightly more important to the overall model fit than contagion (−0.49).

## Discussion

A number of abiotic and biotic factors influence the drift dynamics of fish [54], [55] and invertebrates [56], with passive downstream migration greater at higher discharges [57], [58]. Moore [24] first described the downstream drift of Arkansas River shiner's semi-buoyant pelagic eggs and proposed a relationship between elevated discharge and the onset of spawning. Our study similarly highlights the role of river discharge in driving the timing of the peak in gellan bead catches. The sites with higher discharges on the Canadian River had a greatly reduced median time to peak gellan bead captures compared to those in the North Canadian River where discharge was much lower. Transport velocities in our study were lower than those reported in other studies (0.7 m/s: [36]; 0.57–1.07 m/s: [38]). However, the direction of the response is consistent with similar egg surrogate experiments in the Rio Grande and Pecos River, where transport time was highly positively correlated to river discharge [36].

While the median gellan bead capture time was only correlated with discharge, the length of sampling period was also related to habitat complexity, particularly the dispersion and interspersion of habitat patches within the landscape [40]. Variation was high among sites relative to the length of time required to capture the bulk of the gellan beads but there was no significant relation to the timing of the peak captures. As discharge and contagion index increased, the length of time taken to capture the majority of the beads decreased. A lower contagion score suggests a landscape consisting of multiple small and dispersed habitat patches [39]. Spatial arrangement of patches is important in structuring the downstream dispersal of passive drifting particles [59]. Within the context of the Great Plains rivers, lower contagion scores would equate to reaches with interspersed areas of shallow and deep water and in channel features such as sandbars and islands. In the Great Plains, fragmentation linked to water supply reservoir construction has resulted in channel narrowing of braided downstream reaches [60], [61], creating potential for increased particle transport rates. However, the effect of reduced discharge related to the presence of dams may somewhat offset the increased egg transport associated with this reduced habitat complexity. Changes in other aspects of the natural flow regime e.g., timing and variability [62], should also be considered. For pelagic-broadcast spawning cyprinids, increased habitat complexity may reduce downstream transport distance of ichthyoplankton [36], [37]. Dudley and Platania [36] found transport velocities were greatest in narrow and incised reaches of the Pecos River and Rio Grande. They suggested slower transport rates in reaches with wider and more braided channel morphology would allow more time for developing eggs and larva to reach their free-swimming stage [36]. The link between discharge and habitat complexity has also been proposed by Medley *et al.* [37] although they suggested that as discharge increased a greater proportion of beads would be retained in upstream reaches due to increased lateral connectivity and channel storage, see also [38]. However a critique and re-analysis of the Medley *et al.* [37] approach indicated a number of methodological uncertainties and suggested the relationship between width∶depth ratio and retention was ‘weak’ [63].

**Figure 3:**
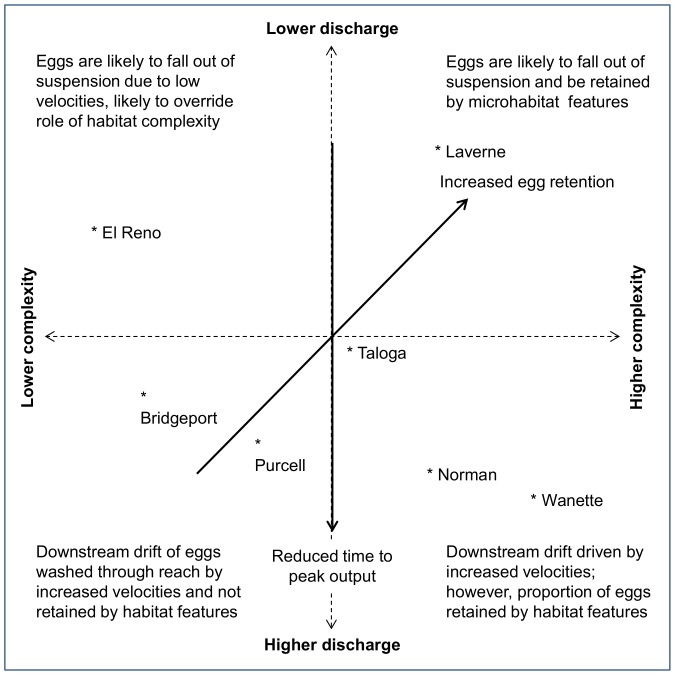
Conceptual framework for the role of hydrology and geomorphology in structuring downstream particle drift. The transport speed and retention of passively drifting particles is determined by the interaction between discharge and habitat complexity. Position of sampling sites is relative rather than absolute.

This study highlights the interaction between hydrology and geomorphology in influencing the distribution of downstream drifting gellan beads and by extension, the eggs of pelagic-broadcast spawning cyprinids. We present a conceptual framework (Figure 3) of how discharge and habitat complexity may interact to influence retention of gellan beads and overlay the relative position of sampling locations to highlight among site variation in geomorphology and hydrology from our study. Discharge determined the median capture time, while discharge and habitat complexity in tandem influenced the retention of beads within a reach. Dam construction has led to a reduction in mean annual discharge in many Great Plains rivers [15]. At very low discharges, gellan beads or fish eggs would likely fall out of suspension [12], [64]. However, gellan beads appear to be more influenced by microhabitat features such as ridges in the sand substrate at discharge levels sufficient for downstream transport but where particles travel low in the water column, (e.g. Laverne site, pers. obs.). This greater retention observed at Laverne does not, however, necessarily equate to increased egg survival (see below). As discharge increased, gellan beads were retained higher in the water column [48] and timing of peaks at a set distance downstream are likely to be reduced. In reaches with low habitat complexity, discharge was the controlling factor (e.g. El Reno, Bridgeport); however, reaches with greater complexity may serve to retain a larger portion of particles in upstream areas (e.g. Wanette, Taloga). High discharges during flood events may transport particles considerable distances downstream, although a positive feedback mechanism may occur whereby lateral connectivity with the floodplain is increased allowing access to low-velocity habitats and thus retaining greater numbers of eggs upstream [37], [38]. While reproductive success is believed to be zero when low discharge reduces Great Plains rivers to a series of isolated pools [32], it has been suggested that floodplains and slackwater areas may provide important nursery habitat for pelagic-broadcast spawning cyprinids [31], [65]. However, within the lower Canadian basin, reduced streamflow associated with reservoir construction has resulted in a reduction in overbank flow and channel narrowing through vegetation encroachment [66].

Our research demonstrates the link between downstream movement of gellan beads, discharge and habitat complexity; however, several factors pertinent to the persistence of populations of pelagic-broadcast spawning cyprinids were not included in our models. How does individual egg behaviour effect downstream transport distance? All eggs are not created equal. Striped bass (*Morone saxatilis*) eggs show between population variation in physical properties, including buoyancy, in relation to the energy in the system [67]. Dudley and Platania [17] showed small levels of variation in the specific gravity (SG) of pelagic-broadcast spawning cyprinids eggs (SG = 1.00589±0.00011). It is therefore likely that individual eggs will respond to the same stimulus differently. Critically, the use of egg surrogates makes it impossible to elucidate the link between downstream drift dynamics and ichthyoplankton survival. The use of gellan beads is driven by the logistics of obtaining large quantities of eggs without impacting populations of threatened species [47]. The release of semi-buoyant eggs is thought to render the eggs less vulnerable to suffocation or abrasion by the sand substrate of the river [68], thus a certain water velocity is required to keep the eggs higher in water column [48]. However, the exact relationship between contact with the substrate and egg viability is unknown. Other factors are also known to affect egg behavior. For example, median buoyancy is negatively correlated with temperature and positively correlated with total suspended solids [64]. However, the sample size within our study was relatively small (seven sites); therefore, results should be interpreted with caution particularly in relation to overfitting of the sampling-period model. Future studies addressing the long-term viability of eggs retained in low-velocity areas e.g., [38], would allow more robust calculations of channel length needed to sustain populations, although tracking such small particles over great distances would be extremely challenging and likely require a combination of laboratory, field and modelling approaches.

Our results highlight how disturbance of the natural functioning of river systems, such as the balance between hydrologic and geomorphologic processes is likely to have contributed to the decline of species like Arkansas River shiner. Anthropogenic activities have altered the natural flow regime and reduced geomorphic complexity of rivers, potentially increasing the length of channel required for ichthyoplankton to reach the free-swimming stage [36]. Possible management options for species such as Arkansas River shiner include dam removal [7], restoring components of the natural flow regime [36] and maintaining perennial base flows [69], which may re-establish channel forming processes [62]. Where appropriate, habitat restoration could enhance habitat complexity and connectivity, thereby increasing egg retention [38].
